# Correction: Shaham et al. Advances in Targeted and Chemotherapeutic Strategies for Colorectal Cancer: Current Insights and Future Directions. *Biomedicines* 2025, *13*, 642

**DOI:** 10.3390/biomedicines13112576

**Published:** 2025-10-22

**Authors:** Salique H. Shaham, Puneet Vij, Manish K. Tripathi

**Affiliations:** 1 Medicine and Oncology ISU, School of Medicine, The University of Texas Rio Grande Valley, McAllen, TX 78504, USA; salique.shaham@utrgv.edu; 2 South Texas Center of Excellence in Cancer Research, School of Medicine, The University of Texas Rio Grande Valley, McAllen, TX 78504, USA; 3 Department of Pharmaceutical Sciences, St. John’s University, 8000 Utopia Parkway, Queens, New York, NY 11439, USA; puneet.vij08@outlook.com

**References**

In our review paper [1], titled “Advances in Targeted and Chemotherapeutic Strategies for Colorectal Cancer: Current Insights and Future Directions”, one of the cited references, reference number 129 (Wang, Z.; Banerjee, S.; Li, Y.; Rahman, K.W.; Zhang, Y.; Sarkar, F.H. Down-regulation of Notch-1 inhibits invasion by inactivation of nuclear factor-κB, vascular endothelial growth factor, and matrix metalloproteinase-9 in pancreatic cancer cells. *Cancer Res.* **2006**, *66*, 2778–2784), was retracted. After review, we noticed that Reference 130 contains the same key information and adequately covers the relevant paragraph. Since Reference 130 serves the same purpose and is a valid source, we kindly ask for the removal of Reference 129 from the manuscript to ensure the accuracy of our references.

With this correction, the order of some references has been adjusted accordingly. The authors state that the scientific conclusions are unaffected. This correction was approved by the Academic Editor. The original publication has also been updated.

## References

[1] Shaham S.H., Vij P., Tripathi M.K. (2025). Advances in Targeted and Chemotherapeutic Strategies for Colorectal Cancer: Current Insights and Future Directions. Biomedicines.

